# Clinical Trials Targeting Secondary Damage after Traumatic Spinal Cord Injury

**DOI:** 10.3390/ijms24043824

**Published:** 2023-02-14

**Authors:** Zin Z. Khaing, Jessica Y. Chen, Gevick Safarians, Sohib Ezubeik, Nicolas Pedroncelli, Rebecca D. Duquette, Tobias Prasse, Stephanie K. Seidlits

**Affiliations:** 1 Department of Neurological Surgery, University of Washington, Seattle, WA 98195, USA; 2 Department of Bioengineering, University of California Los Angeles, Los Angeles, CA 90095, USA; 3 Department of Biomedical Engineering, University of Texas at Austin, Austin, TX 78712, USA; 4 Department of Orthopedics and Trauma Surgery, University of Cologne, 50931 Cologne, Germany

**Keywords:** biomaterials, spinal cord injury repair, acute and sub-acute strategies, neuroprotective therapies

## Abstract

Spinal cord injury (SCI) often causes loss of sensory and motor function resulting in a significant reduction in quality of life for patients. Currently, no therapies are available that can repair spinal cord tissue. After the primary SCI, an acute inflammatory response induces further tissue damage in a process known as secondary injury. Targeting secondary injury to prevent additional tissue damage during the acute and subacute phases of SCI represents a promising strategy to improve patient outcomes. Here, we review clinical trials of neuroprotective therapeutics expected to mitigate secondary injury, focusing primarily on those in the last decade. The strategies discussed are broadly categorized as acute-phase procedural/surgical interventions, systemically delivered pharmacological agents, and cell-based therapies. In addition, we summarize the potential for combinatorial therapies and considerations.

## 1. Introduction

The World Health Organization estimates that 250,000–500,000 people suffer a new spinal cord injury (SCI) each year [1]. Historically, there have been about 17,810 new cases every year in the United States (US) alone [1]. The leading causes of SCIs are vehicular accidents, falls, violence, sports accidents, and medical/surgical complications [1]. Of SCIs in the US, almost 50% are incomplete tetraplegia, followed by complete paraplegia, incomplete paraplegia, and complete tetraplegia [1]. Paraplegia refers to neurological deficits, ranging from minor to complete loss of sensory and motor functions of the lower extremities only, while tetraplegia affects all four extremities. In 2020, the lifetime cost of healthcare for SCI patients was estimated to be USD 1.7–5.1 million for an injury occurring at 25 years of age and USD 1.2–2.8 million for an injury at 50 years of age [2]. While no therapies exist that can reliably restore lost spinal cord functions, extensive research efforts have led to clinical trials of a number of promising treatment options. This review summarizes SCI therapies evaluated in clinical trials in the last decade and some preclinical studies of therapies with a high potential for clinical translation. We focus on therapies administered with the goals of reducing secondary injury and increasing neuroprotection.

Primary injury to the spinal cord is typically caused by a mechanical insult and most often presents as an incomplete injury rather than total severance of the cord. Secondary injury, during which progressive losses of tissue and function often occur, includes acute (within 2 days of primary SCI), subacute (2–30 days after SCI), and intermediate (30 days to 6 months after SCI) phases. Secondary injury is marked by swelling and edema at the site of injury, additional hemorrhage, and an active inflammatory response that leads to extensive death and demyelination of neurons [3]. Around 6 months after primary injury, the SCI is typically considered to have stabilized and be in the chronic phase [4]. Chronic injuries often include fluid-filled cysts surrounded by fibrotic and gliotic scar tissue at the lesion core. Although injury-induced tissue reorganization and remodeling reaches a relatively steady state by the chronic phase, unresolved inflammation is thought to continue for many years [5,6].

Clinical therapies that successfully modulate the inflammatory response to mitigate the detrimental effects of secondary injury after SCI are expected to preserve neurological functions, condition the injury site to support regeneration, and improve patient outcomes. Therapeutic strategies aimed at neuroprotection include (1) early procedural interventions for acute management, (2) pharmacological therapies, (3) cell-based therapies, and (4) combinatorial therapies. Therapeutic agents are being developed to address multiple concurrent processes that occur during secondary SCI, for example, by limiting intraspinal pressure (ISP), modulating the inflammatory response, promoting survival of neurons and their circuitry, myelinating oligodendrocytes, and/or mitigating formation of chronic barriers to tissue regeneration (e.g., scar and cyst formation). Overall, evidence suggests that better clinical outcomes could be achieved with a combinatorial therapy that simultaneously addresses many, if not all, of the issues listed above. Development of therapies that effectively attenuate secondary injury and reduce the barriers to spinal cord regeneration will set the stage for later interventions in intermediate or chronic phases designed to actively promote regeneration of lost tissue. Thus, this review focuses on clinical innovations aimed at limiting secondary injury and promoting neuroprotection.

## 2. Spinal Cord Injury

### 2.1. Intact Spinal Cord

**Figure 1:**
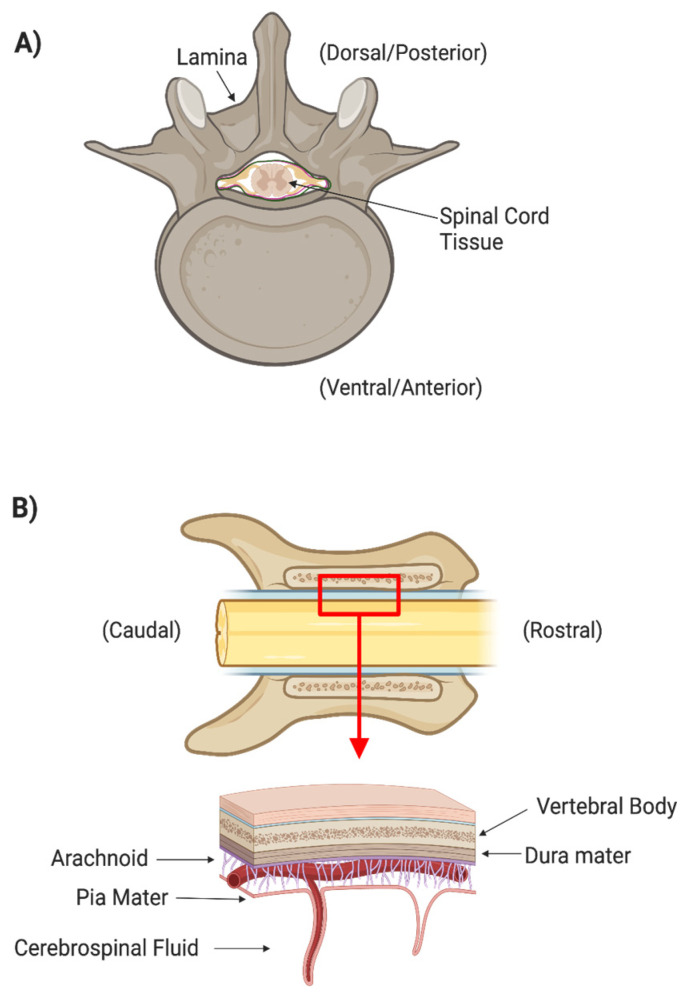
(**A**) Axial cross section of spinal cord surrounded by the boney spinal column. (**B**) Coronal cross section of spinal cord and column showing the different meningeal layers that protect the spinal tissues.

There are many layers of protection that have evolved in the central nervous system (CNS). The spinal cord is protected by the spinal column, which consists of osseous vertebrae. The epidural space between the vertebrae and the outermost meningeal layer contains fat and vasculature to cushion the cord itself from outside mechanical forces (Figure 1). Three meningeal layers provide additional physical protection and separate peripheral extracellular fluids from the cerebrospinal fluid (CSF). The dura mater is the thickest and outermost meningeal layer. Below the dura mater are the relatively thin arachnoid membrane and the innermost meningeal layer, the pia mater. The CSF between the arachnoid and pia mater allows the spinal cord to freely float within the dura mater. The arachnoid is covered in finger-like villi, or arachnoid granulations, which maintain the CSF’s osmotic balance and filter waste from the CSF into the bloodstream. CSF is secreted by specialized ependymal cells in the choroid plexus of the four cerebral ventricles and flows freely through the ventricular-system and central canal of the spinal cord.

Neurons are the primary information carriers of the CNS. All neurons have three main compartments: (1) a cell body (or soma) containing the nucleus and organelles, (2) dendrites that receive inputs from other neurons, and (3) an axon that relays outputs in the form of action potentials. Likewise, glial cells are crucial to CNS function. Oligodendrocytes are glia cells that form insulating, myelin sheaths around axons to facilitate long-range conduction. Areas of dense axonal tracts with insulating myelin appear white in gross CNS tissue and, thus, these areas are commonly referred to as white matter. In contrast, unmyelinated neurons dominate the gray matter of the spinal cord. Astrocytes are glia with diverse roles in the CNS; for example, they provide metabolic support for neurons and form the blood–spinal cord barrier (BSCB) [7,8,9]. Microglia are the resident immune cells of the CNS and are also involved in processes such as synaptic pruning during development [10,11]. Both astrocytes and microglia play important roles in neuroinflammation during the secondary injury response after SCI, as discussed in the following section.

### 2.2. Pathophysiology of SCI

SCI consists of two phases: the primary injury and the secondary injury. The primary injury is the physical force applied to the spinal cord that results in tissue damage. These injuries can be caused by laceration, stretch, compression, and shearing [2]. One of the most immediate consequences of primary injury is the retraction of damaged axons, resulting in loss of these synaptic connections and eventually leading to apoptosis of the damaged neuron and, often, its post-synaptic partner(s) [12,13]. The primary injury also causes local bleeding, loss of physiological blood flow, and edema near the lesion [13]. As components of blood are cytotoxic in the CNS [14,15], limiting parenchymal bleeding in acute SCI is an important strategy to limit secondary injury. Limiting edema and restoring local blood flow through intact vasculature are also important, as ischemia is another major cause of cell death after SCI [13,16].

**Figure 2:**
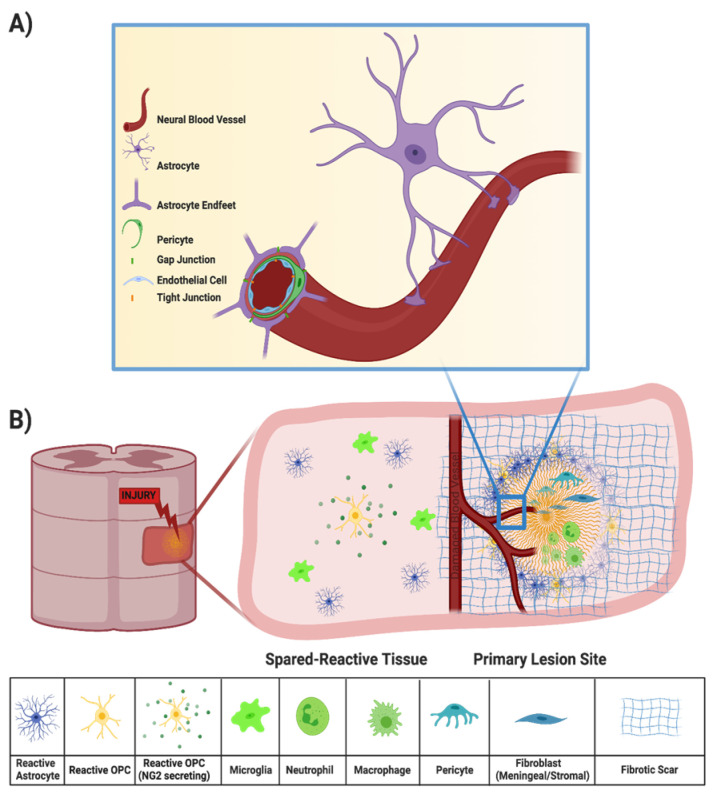
(**A**) Components of the BSCB, in which the neural blood vessel is composed of endothelial cells surrounded by pericytes and end feet of astrocytes that tightly regulate entry of drugs, solutes, and cells into the CNS. (**B**) Components of the secondary injury response, in which a compromised BSCB results in entry of peripheral immune cells, recruitment of microglia, reactive astrocytes, OPCs, and fibroblasts, and fibrotic scar deposition.

Injury to the spinal cord may also disrupt the BSCB, which is created by tight junctions between the end feet of specialized astrocytes and endothelial cells surrounding the blood vessels that supply the CNS (Figure 2A) [9]. All incoming metabolites are shuttled through the BSCB astrocytes, protecting the CNS from chemical compounds and infectious organisms that may otherwise compromise neural functions [9,17]. Peripheral inflammatory cells, such as macrophages and neutrophils, can enter the spinal cord parenchyma from the bloodstream through the compromised BSCB (Figure 2B). Within minutes of the primary SCI, both peripheral inflammatory cells and microglia initiate the secondary injury cascade [18]. While peripheral macrophages and resident microglia both phagocytose cellular debris and potentially infectious agents after injury [19,20], they also secrete a cocktail of pro-inflammatory cytokines that propagate an oxidative inflammatory response that results in additional neuronal death [21,22,23]. Local cytokine concentrations peak during the acute phase of SCI (about 6–12 h after primary injury) but may persist for several days [24,25].

**Figure 3:**
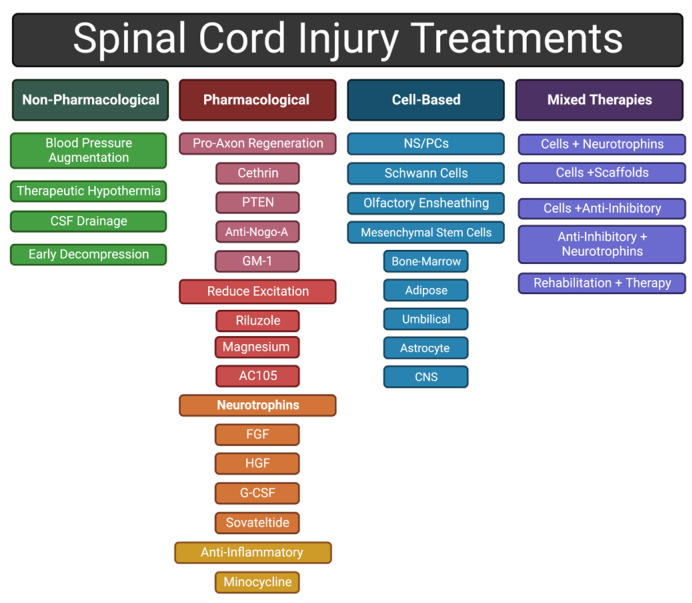
General categories of approaches to treating an SCI discussed in this review.

Debris from damaged axons and myelin sheaths, necrotic/apoptotic cells, and the disrupted extracellular matrix (ECM) also contributes to secondary injury [13]. For example, release of excessive amounts of excitatory neurotransmitters from damaged neurons cause neighboring neurons to fire excessively, leading to excitotoxicity [26]. In another example, high-molecular-weight hyaluronic acid in the ECM is degraded by hyaluronidases after SCI, and loss of interactions between hyaluronic acid and resident astrocytes induces their activation, a fundamental step in glial scar formation [27,28]. Typically, by the end of the subacute phase, the injury center becomes a fluid-formed cyst surrounded by scar tissue and the BSCB has been re-established [4]. Scar tissue includes a glial scar, at the border between injury and intact tissue, and a fibrotic scar, lying between the glial scar and the cyst [29,30]. In this review, we discuss a variety of therapeutic approaches to address the different mechanisms that contribute to the injury response (Figure 3).

The standard clinical assessment of the extent of an individual’s sensory and motor impairments following SCI is the American Spinal Injury Association Impairment Scale (AIS) [31]. AIS A indicates complete disruption of all ascending and descending pathways at a certain spinal segment, such as after complete transection of the spinal cord. AIS B–E indicate increasingly better neurological functions, with AIS E being essentially normal function.

## 3. Managing Acute Pathophysiology after Traumatic SCI

The primary goal of early interventions, typically during the acute phase of SCI, is to reduce tissue loss caused by secondary damage and, thus, preserve more neurological functions for patients. This section discusses the mechanisms of the following investigational, early procedural interventions in greater detail, as well as preclinical and clinical studies evaluating their safety and efficacy in acute SCI with a focus on: (1) elevation of mean arterial pressure (MAP), (2) decompression surgery (DS), (3) CSF drainage, and (4) therapeutic hypothermia (TH). A summary of promising studies for early stage interventions can be found in Table 1.

### 3.1. Augmentation of Mean Arterial Blood Pressure

The most immediate complication in the acute phase of cervical and high thoracic SCI is systemic hypotension, resulting from the disruption of the autonomic nervous system’s regulation of blood pressure [4]. Since 2002, the American Association of Neurological Surgeons (AANS) has recommended that the MAP of traumatic SCI patients be maintained between 85 and 90 mmHg for seven days post-injury [44,45]. In clinical settings, MAP is manipulated with vasopressors such as norepinephrine (NE), phenylephrine (PE), and dopamine [46,47,48,49]. A preclinical study in a porcine model supported the hypothesis that increasing MAP improves blood flow to the injured spinal cord [46]. Specifically, investigators compared the abilities of NE and PE to improve blood flow into an acutely contused thoracic SCI. While both NE and PE increased MAP and improved blood flow and oxygenation at the lesion for a brief period immediately after DS, only PE was associated with a greater possibility of hemorrhage at the SCI site. While these preclinical results suggest that MAP elevation via NE, combined with DS, can safely increase perfusion of the injury site, it remains unclear whether this transient increase in perfusion can affect secondary injury or functional outcomes.

While we have not found any reports from controlled clinical studies, several retrospective studies have investigated whether maintenance of MAP above 85 mmHg during the acute phase of SCI, as recommended by the AANS, had any effects on patient outcomes [50,51]. A 2015 study analyzed San Francisco General Hospital intensive care unit data collected from 100 acute SCIs at all injury levels. MAP elevation was achieved with one or two administrations of NE, PE, and/or dopamine, and some of the patients also underwent DS [50]. Interestingly, the duration of MAP augmentation, defined as the proportion of time that MAP remains elevated, above 85 mmHg was better correlated with neurological recovery than the average MAP over 7 days, a metric that does not account for episodes of hypotension, thereby supporting the need to maintain MAP above a certain threshold for optimal clinical outcomes.

A separate, 2017 retrospective study analyzed the data of 94 traumatic SCI patients who underwent DS early (<24 h after SCI) or late (>24 h after SCI) and were administered NE, as needed, to increase MAP to at least 85 mmHg [52]. Patients for whom MAP was maintained at or above 85 mmHg for at least 2 consecutive hours within 5 days post-injury were at least 10 times more likely to experience improvements in AIS motor grade by 26 days post-injury, independent of when DS was performed. Additionally, a larger proportion of patients who received early DS experienced AIS improvements at later time points (up to 252 days post-injury).

Another smaller retrospective study of 25 traumatic SCI patients, treated at Santa Clara Valley Medical Center in the US, investigated the influence of MAP, measured by an inline arterial sensor during DS, on recovery of motor function [53]. Patients whose MAP remained within the range 70–94 mmHg for longer total periods of time during hospitalization, DS, post-surgery, and acute rehabilitation, experienced greater recovery of motor functions. In addition to supporting the idea that maintaining a normotensive state through pharmacological MAP elevation during the acute phase of SCI increases the likelihood of neurological recovery, this study also suggested that hypertension (MAP >94 mmHg) may have negative effects. Logistic regression analysis in acute cervical SCI patients suggest that increased MAP to the goal of >85 mmHg for 7 days post-injury did not increase the risk for hemorrhagic contusion expansion [54].

In summary, the optimal MAP range may be between 85 mmHg and 94 mmHg. While the duration of MAP elevation during the acute phase of SCI appears to be important, the mechanisms of action for its effects of neurological recovery remain unclear. The clinical use of MAP elevation to treat acute SCI has been rationalized by the idea of a corresponding increase in blood perfusion at the injury site, which is expected to reduce ischemic injury. In a porcine SCI model, MAP augmentation was reported to lead to around a 25% increase in spinal cord blood flow [55]. However, in contrast to this porcine study, there is emerging evidence that impairment in local tissue auto-regulation effectively prevents the expected increase in blood flow, which calls into question whether treatment with drugs intended to augment MAP may benefit acute SCI patients by some alternative mechanism [56]. In the future, it will be important to determine the local spinal vascular mechanisms that can influence adequate tissue perfusion to maximize tissue sparing after SCI.

### 3.2. Therapeutic Hypothermia

TH aims to lower the spinal cord temperature to approximately 33 °C, with the goal of reducing inflammation to limit secondary damage after acute trauma. Systemic TH has been shown to benefit patients in several different clinical situations, including cardiac arrest [57,58], stroke [59,60], traumatic brain injury [61,62], and cerebral aneurysm [63,64]. During acute SCI, there are several potential mechanisms by which TH may be neuroprotective. On the cellular level, lowering temperature reduces basal metabolic rate, reducing energy demands and prolonging survival in ischemic conditions [65,66]. Global hypothermia may also upregulate neuroprotective factors and downregulate inflammatory factors, effectively reducing secondary injury [67,68,69]. Clinically, TH has been realized after SCI either by locally cooling the cord, by administering cold saline to the exposed spinal cord during surgery, or by systemic cooling using a variety of techniques such as cooling blankets, ice baths, or inserting a cooling catheter placed in the inferior vena cava through a femoral vein [65,70].

Several preclinical studies in rodents have found that administering systemic or local epidural TH within 30 min of SCI and prior to DS reduces inflammation, excitotoxicity-mediated secondary injury, lesion volume, loss of neuronal tracts, and locomotor deficits [65,66,70,71,72,73,74,75,76,77,78]. One preclinical study evaluated for how long systemic TH, induced through surface cooling, could effectively mitigate secondary injury from cord compression prior to performing DS [78]. Rodents received TH starting at 30 min and continuing up until 7.5 h after a compression SCI, followed by DS. Rodents who received TH for 7.5 h before DS had significantly better histological and functional outcomes than animals that received only DS 8 h after SCI. Furthermore, rodents receiving TH for 7.5 h before DS compression displayed similar outcomes to those whose cords were compressed for only 2 h prior to DS. Therefore, as TH can be administered *en route* to the hospital, it can be used to mitigate neurological deterioration at the earliest timepoints after SCI before treatments such as DS and CSF drainage are possible.

TH gained widespread attention in 2007 when cold saline was induced intravenously to an NFL player within 15 min of a complete C3/C4 SCI (AIS A) suffered during a televised game. A case report published in 2010 described the details of how systemic TH was applied, the performance of DS approximately 3 h after SCI, and how the patient made an impressive recovery, converting from AIS A to AIS D four months after the initial injury [79]. At least three unique clinical trials since 2010, including around 70 patients, have applied systemic TH administration within an average of 8 h after SCI for durations of 12–48 h and reported that around half of the patients improved by at least one AIS grade within several days of SCI [32,33,34,80]. However, due to the low number of patients and injury heterogeneity, it is not clear whether this level of recovery truly represents improvement compared to the untreated SCI population [80].

Despite its potential benefits for SCI patients, some serious risks are associated with systemic TH, including pneumonia, temperature-related cardiac complications, thromboembolism, infection, and potential insult of the femoral artery [34,35,70]. While application of TH by local cooling in the epidural space above the injury may decrease some of these risks, such as cardiac issues and pneumonia, local and surface cooling raises the risk of infection and variability in cooling. Additionally, some studies have suggested that systemic TH may be more effective at suppressing apoptosis in animal models of SCI [81,82]. One clinical trial investigated effects of local epidural cooling; however, the trial was terminated as three out of five total patients developed infections, a clear risk given the more invasive nature of local cooling [35].

While many side effects of TH remain to be resolved, the benefits are clear. In 2019, the University of Miami completed a prospective, observational study on 41 patients who received systemic TH for 48 h [36]. While results are still pending publication, this same group initiated a larger, multi-center, randomized clinical trial in 2017 [37]. Trial enrollment is still ongoing, and 120 patients are expected by the estimated completion date in 2024.

### 3.3. Cerebrospinal Fluid Drainage

Another approach to reduce mounting ISP in acute SCI is drainage of excess CSF, typically by removing fluid at a constant rate using a syringe inserted into the intrathecal space to steadily lower ISP to about 10 mmHg [83]. In SCI patients, persistent extradural compression can reduce or occlude the subarachnoid space resulting in a CSF pressure differential across the injury site [84]. In a porcine acute contusion model, the cranial and caudal CSF pressures were found to have an increasing pressure differential (0.39 mm Hg/h) during the compression [84]. After decompression, cranial ISP decreased but caudal ISP increased, resulting in no net change in CSF pressure differential. Thus, recording CSF pressure in the lumbar spinal cord will likely not be sufficient to estimate pressures rostral to the SCI.

In acute SCI, it is generally thought that CSF drainage should allow for increased perfusion pressure within the spinal cord. However, a porcine study reported that, unlike MAP augmentation that led to short-term improvements in perfusion, CSF drainage alone did not substantially improve blood flow in the injured spinal cord [55]. Interestingly, performing CSF drainage in combination with MAP augmentation did result in longer term improvements in spinal cord perfusion when compared to MAP augmentation alone.

In human subjects with acute SCI, a phase 1/2 clinical trial in the US, including 22 patients with acute cervical or thoracic AIS A-C SCIs, investigated the safety and efficacy of CSF drainage [38,39]. Around half of the patients underwent CSF drainage within 48 h of SCI followed by ISP recording during the next 72 h. While the average ISP for patients who underwent CSF drainage did not fluctuate significantly, there was a significant rise in the average ISP for patients who did not receive CSF drainage. While no significant behavioral or histological changes were reported as a direct result of CSF drainage, this trial demonstrated procedure safety and motivated a phase 2b clinical trial that included patients with acute (≤24 h of injury) cervical (C4-C8) AIS A-C SCIs [40]. Patients were divided into two cohorts who received either CSF drainage (achieving ISP of 10 mmHg) and MAP elevation using an NE drip (achieving 100–110 mmHg) or MAP maintenance only. This study concluded in August 2019 with 15 patients and the results are pending. It is not clear to the authors of this review why MAP was maintained above the AANS recommended range for acute SCI in this study. While CSF drainage was found to be safe in these trials, it is possible that excessive removal of CSF may cause the spinal cord and brain to lose the cushioning provided by the CSF and, in turn, contact surrounding bony structures, leading to temporary neurological deficits [85,86,87]. Other potential complications of the drainage procedure include leakage of CSF at the site of dural puncture, subdural hematoma, cerebral herniation, and infection [83,88,89].

### 3.4. Decompression Surgery

Following primary SCI, inflammation leads to increased ISP, resulting in compression of injured tissue, occlusion of local vasculature, and ischemia [90,91,92,93,94]. ISP ranges from 4 to 18 mmHg for healthy individuals to 30–35 mmHg for patients with acute contusive SCIs [95,96]. DS aims to alleviate excessive ISP by increasing the volume available for spinal cord tissue swelling or to remove bony fragments infringing into the spinal cord [97,98]. DS almost always involves a laminectomy, which is the removal of the vertebral lamina, and is often coupled with vertebral instrumentation to restore stability of the spinal column. Several clinical studies have found that performing DS within 24 h of the primary SCI is associated with shorter hospital stays and increased probability of post-operative neurological recovery, while other studies of surgeries within 72 days or later of injury did not report any benefits [41,98,99,100,101,102,103,104,105,106]. In general, studies have found benefits of early DS only for patients with incomplete injuries and AIS B–D, but not with more severe, complete injuries, and AIS A [42,43,107]. However, a systematic meta-analysis of clinical trials did not find statistically significant long-term advantages of early over late DS, which the authors suggested could be due to variability in the level of SCI, follow-up timing, and specific outcomes being measured [107].

Furthermore, cases of extensive edema and swelling may result in deformation of the dura-bound spinal cord [108,109], in which additional decompression may be achieved through opening the meningeal membranes, including the dura membrane (durotomy) alone or in addition to the pia mater (piotomy) [43]. Typically, a duraplasty is performed after durotomy to re-seal the meningeal membrane using synthetic, allogenic, or autologous grafts [110]. Preclinical animal studies demonstrated that meningeal decompression after laminectomy, including durotomy and piotomy, was more effective than laminectomy alone, in terms of reducing ISP, and resulted in improved functional outcomes [94,111]. A human cadaveric study compared the ability of three DS approaches (laminectomy only, laminectomy with durotomy, and laminectomy with durotomy and piotomy) to reduce ISP, measured at maximal kyphosis by sensors placed within the cord throughout the cervical and thoracic segments [112]. On average, this study found that laminectomy alone reduced ISP by around 20%, while midsagittal durotomy or durotomy with piotomy additionally reduced ISP by around 66% and 99%, respectively. Although this study used a cadaveric model that does not account for the dynamic edema of a living subject, these findings provide evidence that meningeal incision reduces ISP to a greater extent than laminectomy alone. However, drastic reductions in ISP following durotomy and piotomy may be detrimental in cases of moderate edema, as ISP could drop below physiological levels.

A small-scale clinical study performed in China measured changes in ISP before and after durotomy in three patients with excessive edema and reported that durotomy followed by duraplasty using a polymeric synthetic graft lowered ISP [43]. While this prior study unfortunately did not have a durotomy-only control group, results from another small-scale study performed in China did observe better recovery of neurological function in patents receiving durotomy with duraplasty when compared to durotomy alone [42,43].

The duraplasty graft used may also have significant effects. Preclinical studies, performed in rodent compressive SCI models, have reported that durotomy followed by allographic duraplasty leads to reduced cystic cavity volumes, inflammation, scar tissue formation, and secondary injury overall [113,114,115,116]. Conversely, in rodent compressive SCI models where synthetic grafts were used, no histological changes were observed, some animals displayed increased lesion sizes, and others had decreased hindlimb coordination as well as the development of neuropathic pain [117,118]. Differences in the effects of allographic and synthetic duraplasty may be due to the presence of specific bioactive factors present only in allografts or biocompatibility issues inherent to the synthetic graft materials used. For example, duraplasty using a hydrogel-based dural sealant, DuraSeal, led to additional compression of the spinal cord in several subjects after the material expanded in vivo [117].

**Figure 4:**
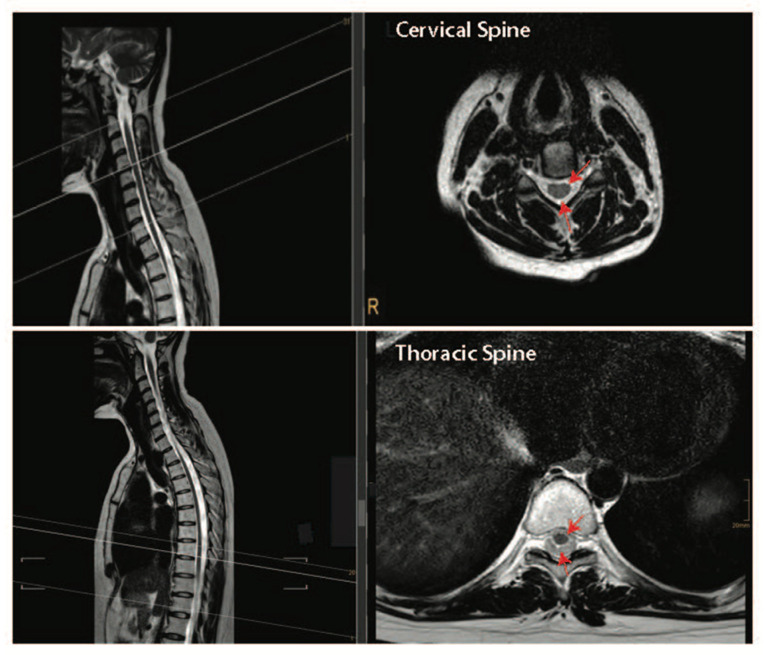
Intact cervical and thoracic spinal cord tissue can float freely within the spinal column in humans. Top panel—T2-weighed sagittal and cross-sectional images of the cervical spine. Bottom panel—T2-weighed sagittal and cross-sectional images of the thoracic spine. Dark structures where the red arrows are pointing are the spinal cord tissue and the lighter areas depict cerebral spinal fluid-filled subarachnoid space. Images are courtesy of Dr. Tobias Prasse.

Together, the pre-clinical and clinical data presented above suggest that performing both a durotomy with a piotomy may be more effective than a durotomy alone at reducing the raised ISP after acute SCI. However, the highly invasive nature of durotomies and piotomies introduces risks, including further mechanical damage to the spinal cord, infections, excessive ISP reduction, CSF leaks and fistulas, hemorrhage, and pseudo-meningoceles, thereby necessitating a thorough risk–benefit analysis that accounts for patient-specific injury pathologies [119]. For example, the risk–benefit analysis for durotomy and piotomy likely depends on the SCI level [112]. Specifically, the human spinal cord tissue occupies only 50% of the subdural space within the thoracic spinal cord, while it occupies about 90% of the subdural space within the cervical spinal cord (Figure 4). Given the larger space in which the spinal cord can expand without dural compression at the thoracic level [120], benefits associated with a durotomy and/or piotomy may be more pronounced for cervical level injuries, where more severe swelling would lead to stronger cord compression. Furthermore, as discussed above, performing DS within the acute phase of SCI (within 72 h) appears to facilitate significant functional recovery [97,100,115,121].

In the future, it will be important to identify specific patients who would benefit from both bony and/or meningeal decompression using intraoperative imaging that can detect local changes in blood flow and tissue perfusion. One such intraoperative imaging technique is in development by Khaing et al., using an ultrafast, contrast-enhanced ultrasound technique to image and quantify acute hemodynamic changes in rat traumatic SCI models [122,123]. Development and use of such intraoperative imaging modalities that can assess local blood flow changes will be essential for effective identification of patients who would likely benefit from invasive DS.

## 4. Pharmacological Therapies

The following sub-sections discuss several pharmacological agents that hold promise for treatment of acute SCI with the goal of mitigating secondary injury by reducing bleeding, inflammation, and excitotoxicity, as well as improving angiogenesis and/or neurogenesis. A summary of promising pharmacologic therapies can be seen in Table 2. While the pharmacological agents targeting barriers of axonal regeneration also hold promise as treatments for acute and chronic SCI, they are beyond the scope of this manuscript. Thus, readers are referred to a recent review of this area by Uyeda and Muramatsu (2020) [124].

### 4.1. Reducing Intra-Spinal Bleeding

Intraspinal bleeding in acute SCI is thought to promote secondary damage and compromise functional recovery [137,138,139,140]. Glibenclamide (also known as glyburide) is a potent blocker of sulfonylurea receptor 1-regulated, calcium-activated, [ATP]-sensitive, nonspecific cation channels, which are regulated in endothelial cells during acute SCI [141]. Systemic treatment with glibenclamide at 200 ng/h delivered via a subcutaneously implanted osmotic minipump may aid in reducing the effects of secondary hemorrhage and progressive hemorrhagic necrosis that follow SCI [142,143]. In pre-clinical studies, multiple laboratories have shown that early treatment with glibenclamide after a cervical-level contusion in rats can prevent capillary fragmentation, reduce progressive hemorrhagic necrosis, reduce lesion volumes, and improve functional outcomes [141,142,143]. As glibenclamide is already an FDA-approved treatment for type 2 diabetes, it is an attractive and low-risk molecule for translation in SCI therapeutics. A phase 1 clinical trial began in 2017 to evaluate the safety of oral glibenclamide treatment of acute cervical SCI patients (AIS A-C) [125]. However, this trial was terminated in 2021 due to low enrollment and the principal investigator leaving the institution [125]. Given evidence from preclinical studies, glibenclamide remains a promising treatment for limiting secondary cell death due to progressive hemorrhage after acute SCI.

### 4.2. Reducing Inflammation

A variety of studies have shown that post-SCI inflammation further exacerbates neuronal damage following the initial mechanical trauma and that reducing inflammation can encourage cell survival, increase regeneration, and decrease muscle denervation [144,145,146,147,148]. While many studies are aiming to better understand how inflammation drives secondary injury, they have relied on experimental techniques that have not yet been readily translated to a clinically setting (e.g., rodent genetic techniques, gene therapy, biomaterials carriers for stem cell transplants) and these studies have overwhelmingly reported that decreasing inflammation benefits functional outcomes [149]. This section discusses several pharmacotherapies that, while approved by the FDA for non-SCI uses, act by decreasing inflammation and, thus, may provide some benefits to SCI patients. In addition, these compounds have been previously evaluated by the FDA for other applications and could potentially be translated to clinical use at a relatively accelerated pace.

Previously, methylprednisolone sodium succinate (MPSS), a synthetic corticosteroid, was used off-label for management of acute SCI. It was thought that, since MPSS and other steroids are known to cause immunosuppression and therefore limit inflammation, these drugs may be useful in the setting of an acute SCI. In recent years, MPSS use in SCI has fallen out of favor, as there is increasing evidence that it can result in serious complications in polytrauma patients, while clinical evidence that MPSS effectively prevents secondary damage is minor [150,151]. While approved for multiple other indications, MPSS never received FDA approval for SCIs.

Minocycline is a semisynthetic, lipophilic, tetracycline-derived antibiotic that can penetrate the BSCB, enabling systemically delivered drugs to reach the CNS. In vitro models suggest that minocycline inhibits the expression of pro-inflammatory cytokines to prevent neurotoxicity and apoptosis [152]. Several preclinical studies in rodent models of acute SCI have collectively demonstrated that minocycline induces increased expression of anti-inflammatory cytokines (i.e., IL-10), decreased expression of several proinflammatory cytokines (i.e., NO, TNF-α, IL-1β, and IL-6), while inhibiting recruitment and activation of microglia [153,154,155,156]. Together, these actions effectively dampen the inflammatory response, resulting in markedly improved functional outcomes and smaller lesion sizes.

A phase 2 clinical trial in Canada evaluated the efficacy of intravenously delivered minocycline to patients with cervical or thoracic SCI within 12 h of injury and continued for 7 days [126,127]. Patients additionally underwent DS within 24 h of primary injury. Strikingly, patients with cervical injuries given minocycline experienced an average 14-point improvement in motor score, while patients with thoracic injuries given minocycline experienced no significant difference in functional recovery when compared to patients in the control group with DS only. While the study had a relatively small sample size, results suggest that minocycline therapy may be beneficial to SCI patients suffering from cervical SCI. These findings motivated a phase 3 trial, initiated in 2013, which used the same paradigm for treatment as the phase 2 trial, but focused on patients with acute cervical SCI (AIS A-D) [128]. The study was expected to reach completion in 2018, but its status has not been updated since 2014 and the current status is unknown.

Like minocycline, azithromycin has been approved by the FDA for use as an antibiotic drug. Beyond its antibiotic function, azithromycin can induce peripheral macrophages, which readily infiltrate lesions in acute SCI, to adopt a pro-regenerative phenotype, sometimes characterized as M2 macrophages [157,158]. Studies in vitro show that azithromycin reduces the expression of pro-inflammatory cytokines (i.e., IL-12, IL-6, IL-1β, TNF-α, and NO) [90,159,160,161,162,163]. Preclinical studies using mouse models of thoracic contusion have demonstrated that azithromycin treatment for 7 days, starting within 30 min of SCI, increased the presence of M2-type macrophages near lesions in a dose-dependent manner and led to improved tissue sparing and recovery of locomotor function [159,164].

More recently, studies in a rat thoracic contusion model showed that azithromycin delivered at 30 min post-SCI and continued daily for 3 days results in significantly improved locomotion, decreased mechanical sensitivity, and decreased thermal allodynia [165]. In addition, this study corroborated previous in vitro and in vivo results [157,158,159,160,162,164], demonstrating that azithromycin treatment significantly altered the lesion microenvironment, as evidenced by decreased levels of TNF-α, increased levels of IL-10, increased numbers of M2 macrophages, and decreased presence of M1 macrophages. Although no clinical trial data are available, this shift from a pro-inflammatory to a pro-regenerative microenvironment has been associated with improved outcomes in SCI patients [159,164,165]. Given the strong preclinical evidence and previous FDA approval, azithromycin is ripe for translation to clinical trials for treatment of acute SCI.

### 4.3. Reducing Neuroexcitation Toxicity

Following an SCI, excitatory neurotransmitters released from damaged axons increase electrical activation of adjacent neurons, flooding their intracellular compartments with ions such as Na^+^ and Ca^2+^ [166]. This influx of excitatory species induces rapid firing and ultimately leads to neuronal cell death, in a process referred to as excitotoxicity [166,167,168]. Another mode of excitotoxicity stems from the injury causing metabolic changes and membrane damage, such that a neuron’s membrane potential can no longer be appropriately regulated. As a result, excess intracellular Ca^2+^, which broadly activates calcium-dependent enzymes with widespread downstream activities, then leads to structural and functional damages within the neuron [169]. This section discusses small-molecule drugs thought to confer neuroprotection through reduction in excitatory neurotoxicity, one of the major drivers of secondary injury following SCI.

Riluzole is a benzothiazole currently FDA-approved for the treatment of amyotrophic lateral sclerosis (ALS). Riluzole has many potential mechanisms of action, but generally functions to decrease neuroexcitation [170,171,172,173]. In a rodent model of compressive cervical SCI, daily riluzole administration for 7 weeks after injury improved motor recovery and attenuated neuropathic pain at 8 weeks after injury when compared to control animals [174]. Further analysis revealed decreased activation of the NMDA (N-methyl-D-aspartic acid) receptor for the neurotransmitter glutamine in astrocytes near lesions in riluzole-treated animals, suggesting that astrocytes experienced less glutamatergic overexcitation. Furthermore, riluzole treatment diminished microglia activation, which the authors posit may be a result of protection of neurons and glia from excitotoxicity and apoptosis. Despite these positive results, a separate study compared therapeutic effects of riluzole, TH, and glibenclamide, each administered 4 h after cervical contusion in rodents, and found that, while riluzole was beneficial, both TH and glibenclamide were more effective at decreasing lesion volume and mitigating the extent of motor deficits [175].

Given the relatively large differences in responses to SCI and the immune systems between rodents and humans, effects in human trials may be very different [176]. A prospective multicenter phase 1 trial completed in April 2012 was the first in the US to administer riluzole to SCI patients, including both cervical and thoracic injuries [131]. Patients with cervical SCI who received riluzole orally every 12 h for 14 days after SCI experienced robust improvements in functional grades that exceeded historical patient data [129]. While these benefits were not observed in thoracic SCI patients, this may be due to the low enrollment rate of these patients. Interestingly, when compared to data from ALS patients, SCI patients had substantially lower effective plasma concentrations of riluzole, indicating that the administered dosage may need to be increased to maintain an effective dose [177]. If benefits for SCI are dose-dependent, treatment efficacy may be improved with higher doses of riluzole [130]. These exciting findings led to a multicenter, randomized, placebo-controlled, double-blinded, phase 2/3 clinical trial of riluzole for acute cervical SCI in the US, initiated in October 2013 [132,133]. Unfortunately, the trial was terminated early due to poor enrollment and no results have been published.

### 4.4. Biologics Promoting Angiogenesis and Neurogenesis

Many researchers have posited that an effective treatment for SCI will require a combination of actions, including promoting vascularization and inflammatory resolution [149,178]. Blood vessels in the spinal cord, compromised by the initial physical SCI and subsequent edema, create an ischemic environment, and contribute to secondary injury. Sovateltide, also known as IRL-1620, SPI-1620, or PMZ-1620, is a synthetic endothelin-B receptor agonist used to combat ischemic injury. Activation of endothelin-B receptors, expressed by vascular endothelium, induces secretion of nitric oxide, which causes vasodilation [179,180]. In a rodent model of cerebral stroke (middle cerebral artery occlusion), sovateltide administered in the acute phase of injury enhances angiogenesis, neuronal survival, and neurogenesis, while reducing oxidative stress [180,181,182]. Together, these effects are likely responsible for the observed reductions in infarct size and loss of neurological functions [181].

Promising results in preclinical studies have motivated human trials. An interim report of a recently completed prospective, multicentric, randomized, double-blinded, placebo-controlled clinical trial run by Pharmazz, Inc. in India found that sovateltide, administered as an intravenous bolus at 1, 3, and 6 days after an acute cerebral ischemic stroke, was well tolerated and significantly improved patient outcomes [183,184]. Pharmazz, Inc. is now recruiting for a phase 2 trial in India evaluating effects of an equivalent dosing regimen of sovateltide after acute injury, specifically for partial SCI (C5-S5) where no vertebral fracture has occurred [134].

Granulocyte colony stimulating factor (G-CSF), also known as filgrastim, is a growth factor. It is used clinically as a pegylated version of recombinant human G-CSF, produced in a mammalian cell system, to stimulate white blood cell production. In vitro, G-CSF has been shown to protect primary cortical neurons against excitotoxicity induced by glutamate [185]. In various animal models of CNS injury, G-CSF has been found to stimulate angiogenesis and neurogenesis, reduce inflammation and scar formation, and improve tissue sparing, presumably leading to functional recovery [186,187,188,189,190]. A series of clinical trials in Japan determined that an intravenous dosage of 10 μg/kg/day of G-CSF within 48 h of cervical or thoracic SCI and continuing for 5 consecutive days was safe [191]. At a one-year follow-up, 15 out of the 17 patients who received G-CSF experienced improvements to their overall AIS, compared to only 9 out of 24 control patients [192]. However, patient cohorts were determined based on the institute in which the patients were treated, rather than in a random manner, and control patients did not receive placebo injections [192]. More recently, the same research group published results of a prospective, multicenter, randomized, double-blinded, placebo-controlled comparative study of G-CSF treatment for acute cervical (C4-C7) SCI (AIS B and C) in 26 patients, reporting no significant differences in functional improvement between patients in the G-CSF and control groups 3 months after SCI [136]. However, they did report a trend towards better outcomes in patients who received G-CSF 6 months after SCI, indicating that G-CSF may have some late-term benefits [135,193].

## 5. Cell-Based Therapies

Many studies have focused on developing cell-based therapies for SCI, several of which have been investigated in clinical trials of patients with acute and chronic SCI. Among these are multipotent stem cells and differentiated glia. While cell therapies may benefit SCI repair in several ways, the expected beneficial mechanisms can be categorized into two broad categories: (1) mitigation of secondary injury through secretion of neurotrophic and anti-inflammatory factors, which improves tissue sparing and establishes an environment conducive to regeneration, and (2) remyelination and functional engraftment into host neural circuitry, which represents true tissue regeneration. While the latter goal of tissue regeneration is obviously highly desirable and may be necessary to adequately treat chronic SCI, many barriers remain for translation of this strategy, including poor engraftment, extensive apoptosis, and inappropriate differentiation with potential for tumorigenesis of transplanted cells [194,195]. In contrast, leveraging the anti-inflammatory properties of the secretome of cells transplanted into an acute SCI environment likely will be easier to translate to clinical practice, given that these cells only need to live for a relatively short period of time and do not need to differentiate or form functional synaptic connections to be therapeutically effective. The latter goal is the focus of this section.

Researchers have explored transplantation of cells sourced from various tissues, at various stages of differentiation, and treated with various ex vivo protocols to identify cell sources and biomanufacturing methods that produce effective therapeutics for acute SCI. While autologous sources, such as from induced pluripotent stem cells (iPSCs), Schwann cells (SCs), or mesenchymal stem cells (MSCs), would avoid potential complications from immune-rejection, variation of these cells across patients and the economic costs of personalized biomanufacturing are difficulties that may be avoided by development of a single, therapeutic cell line from an allogeneic source, such as neural stem/progenitor cells (NS/PCs) derived from an established embryonic stem cell line. Furthermore, autologous cell sources may not be feasible for older individuals, whose cells are likely less proliferative and may not retain sufficient plasticity. Culture, expansion, and maintenance of therapeutic cells also present challenges. For example, culture methods must eliminate animal products, such as serum, to avoid immunogenicity after cell transplantation [196,197,198].

Additional challenges include the timing, injection location relative to the injury, and method of stem cell delivery. For example, earlier administration (e.g., within 48 h of primary SCI) may be more effective, but the preparation of autologous cells is time intensive. The location and method of transplantation are both key determinants of how well transplanted cells survive. Cells injected into the spinal cord tissue adjacent to, rather than directly into, lesions or within biomaterial carriers [199,200,201] had improved survival rates. This section focuses on therapeutic stem cells, derived from a variety of sources, that have been investigated in clinical trials for their ability to reduce secondary injury when used to treat acute SCI. A summary of promising cell-based therapies can be seen in Table 3.

### 5.1. Mesenchymal Stem Cells

MSCs are self-renewing, multipotent cells that can be derived from multiple sources, including umbilical cord (UC-MSCs) or bone marrow (BM-MSCs). MSCs are typically associated with their capacity to generate cells comprising connective tissues, fat, skeletal and cardiac muscle, and cells of the immune system, such as macrophages [233,234]. However, given the proper cues, they can also readily differentiate into neurons and/or glial cells in vitro [235,236] and in vivo [237,238]. MSC administration in cases of acute or subacute SCI has been found in phase 1 clinical trials to be well tolerated in both the short and long terms [239,240,241,242]. In general, MSCs are given in three or four doses over 1–12 weeks in clinical trials. With regard to treatment efficacy, several studies have reported notable benefits to individual patients. However, two recent meta-analyses of human trials found modest improvements in AIS sensory scores, but not AIS motor scores [242,243].

The therapeutic benefits of MSCs have been attributed primarily to secretion of anti-inflammatory and proregenerative proteins and extracellular vesicles near the injured tissue, rather than to the differentiation of transplanted MSCs into new, functional tissues [244,245]. Therapeutic MSCs have been administered via intrathecal, intraspinal, and intravenous injection [246,247]. While intrathecal and intraspinal injections likely result in a greater number of transplanted cells reaching the injury site, intravenous delivery is less risky and invasive for the patient. However, intravenous delivery requires MSCs to travel from the systemic circulation, through the BSCB, into the injury site. While there is abundant evidence that MSCs respond to factors secreted in injured tissue and will travel towards the injury in response, intravenous administration may be limited by the BSCB. As the BSCB heals, it may prevent MSC entry into the CNS [248].

MSCs can be used for autologous (isolated directly from the patient) or allogeneic (isolated from a separate individual) transplantation. For autologous use, UC-MSCs would need to be banked at birth for later in life, while BM-MSCs can be isolated from adult patients at the time of need. MSCs can also be derived from adipose tissues (AD-MSCs) for autologous transplantation. While we have not found any clinical trial results evaluating administration of AD-MSCs in patients with subacute SCI, there are ongoing phase 1 [203] and phase 2 [204] clinical trials in the US for chronic SCI with estimated completion dates in 2023 and 2024, respectively. Initial reports of the patients from the phase 1 trial showed no severe adverse events associated with AD-MSC delivery at 3-, 6-, 12-, and 18 months post-injection [202,203]. In a rat model of contusive, thoracic-level SCI, AD-MSCs or UC-MSCs were transplanted intrathecally during the subacute phase of injury. Both types of MSCs provided similar benefits by modulating the immune response to be more amenable to axon regrowth [249]. However, AD-MSCs and UC-MSCs had unique cytokine and gene expression profiles, suggesting they differ in their mechanisms of action.

#### 5.1.1. UC-MSCs

UC-MSCs are non-invasively and readily accessible immediately after birth in the Wharton’s jelly, a gelatinous tissue within the umbilical cord [250]. Autologous transplantation may be a viable strategy in the future if UC-MSC isolation and banking become standard practice. Allogeneic sources are also reasonable, given that UC-MSCs exhibit limited immunogenicity through low expression of MHC type II and co-stimulatory molecules [251,252,253]. While previously the umbilical cord was considered as waste tissue after birth, ethical concerns with allogenic transplantation eventually may require a similar infrastructure to organ donation. Notably, UC-MSCs have secretomes whose composition is unique from that of MSCs sourced from other tissues. UC-MSCs have a higher proliferation capacity and potentially lower immunogenicity when compared to other MSC sources [251,253,254] and are thought to induce a shift from a proinflammatory tissue microenvironment to a pro-regenerative one [255,256,257,258]. Together, the immunomodulatory properties of UC-MSCs make them an attractive therapeutic candidate for acute SCI, where they would be expected to reduce the negative effects of secondary injury.

A handful of small-scale clinical trials in China have established the safety and, tentatively, efficacy of UC-MSC transplantation into the acutely injured spinal cord, e.g., intrathecally or under the arachnoid membrane [205,206,259]. No adverse events have been reported, and several patients experienced sensory or motor improvements after treatment. A recently completed randomized, double-blinded, placebo-controlled, phase 1/2a clinical trial [209] in Spain showed that intrathecal UC-MSCs can improve pinprick sensations in dermatomes below the injury level when compared to controls [208]. While most completed clinical trials have focused on patients with chronic SCI and do not provide any information on how UC-MSCs may provide immunomodulation in acute SCI to mediate recovery, one phase 1/2 clinical trial giving UC-MSC injections monthly for 4 months in subacute or chronic cervical, thoracic, or thoracolumbar SCI patients resulted in dramatic improvements in motor control, bowel/bladder function, and light touch sensation that had plateaued by 12 months after final administration, with only mild adverse events such as fevers and headaches after injection [206,207]. Another phase 2 clinical trial to confirm these prior results in subacute SCI is currently recruiting, although the status of this trial has not been updated since April 2019 [210].

Recently, Xiao et al. (2021) demonstrated that injection of extracellular vesicles secreted by UC-MSCs into a T10 clip compression SCI in a rodent model decreased astrocyte activation, increased neuronal sparing, and improved recovery of motor functions [244]. They further found that repair was mediated by miR-29b-3p released from extracellular vesicles, which inhibited PTEN activation to upregulate the Akt/mTOR pathway. Use of extracellular vesicles, rather than the original UC-MSC source, is an attractive route towards cell-free therapies that could replace cell transplantation.

#### 5.1.2. BM-MSCs

Bone marrow is a rich source of MSCs in adults. Bone marrow transplantations have been standard clinical practice for decades for the treatment of immunological diseases, including leukemia, establishing that BM-MSCs are safe for human transplantation. However, bone marrow harvesting is an invasive procedure, especially if compared to procedures such as UC-MSC isolation. A phase 1 clinical trial in Pakistan has demonstrated safe repeated administration (typically two to three doses within a one-month span) of autologous BM-MSCs through intrathecal injections in subacute and chronic SCI patients [213]. While the study sizes were small and without placebo controls, several individual patients showed slight-to-modest improvements in motor and sensory functions in follow-up examinations, which typically took place 6–12 months after treatment without any treatment-related adverse events [212]. Currently, there are two phase 1 clinical trials in Jordan, which compared effectiveness for SCI treatment of intrathecal injection of BM-MSCs to that of AD-MSCs [214] or UC-MSCs [215] with results pending.

Despite promising results, efficacy of BM-MSC therapy has not been widely demonstrated. However, it is apparent from the pilot and phase 1/2 clinical data available that BM-MSC administration may be most beneficial when performed during acute and subacute, rather than chronic, SCI cases. In this study, most subacute SCI patients showed motor and sensory improvements 3 months after cell delivery, while only 1 out of 13 chronic SCI patients showed improvements [211,260]. Thus, BM-MSC secretion of immunomodulatory and neurotrophic factors, which can reduce effects of secondary injury, may be responsible for these benefits rather than by true regeneration. In the future, timing and method of BM-MSC administration will likely require further optimization to yield greater and more consistent benefits for SCI patients.

Athersys, Inc. has developed a BM-MSC-derived cell product of multipotent adult progenitor cells (MAPCs) for allogenic transplantation, termed MultiStem^®^ MAPCs, which represent the non-hematopoietic stem cells present in bone marrow stroma. Compared to BM-MSCs, MAPCs have a broader differentiation potential and are less susceptible to senescence in culture [261,262,263]. Phase 1 and 2 clinical trials have shown intravenous infusion of MultiStem^®^ to be both safe and effective in cases of ischemic stroke [264,265]. Therapeutic effects were greater when cells were administered closer to the time of ischemic injury, indicating these effects were due to mitigation of secondary injury. While there have yet to be clinical trials exploring MAPCs for SCI, Depaul et al. investigated intravenous administration of MAPCs immediately following a moderate, contusive SCI at T8 in rats and reported significant gains in motor function with MAPC treatment [266]. However, they also reported that few MAPCs were found in the spinal cord and that most actually trafficked to the spleen. Thus, it is possible that intravenously delivered cells induce a peripheral immune response that limits inflammation in the CNS, similar to the mechanism by which acutely and intravenously administered synthetic nanoparticles have been shown to improve recovery after SCI [267].

### 5.2. Neural Stem/Progenitor Cells

NS/PCs, which have the potential to differentiate into all cell types present in the adult CNS, are often categorized by their degree of plasticity. NS/PCs include multipotent cells, which can become neurons or glia, and lineage-restricted neural or oligodendroglial progenitors. Therapeutic NS/PCs can be obtained from both allogenic sources, as with cell lines generated from fetal brain or spinal cord tissue or in vitro differentiation of an established ESC line, and autologous sources, as with iPSC-derived NS/PCs. Several researchers have explored the ability of NS/PCs to regenerate tissues in chronic SCI in animal models [268,269,270,271,272]. These studies aimed to replace lost neural tissue or develop relay circuits by transplanting NS/PCs. However, this section focuses on the use of NS/PCs to modulate the neuroinflammatory environment during acute SCI to improve functional recovery. Like MSCs, NS/PCs secrete an assortment of factors with immunomodulatory and neuroprotective effects [273,274,275], making NS/PC administration during acute SCI an attractive strategy to reduce secondary injury [276,277].

#### 5.2.1. Multipotent Neural Stem/Progenitor Cells and Neuronal Progenitor Cells

Several preclinical animal studies have reported that NS/PCs transplanted during subacute SCI reduces inflammatory activation of glial and immune cells [205,274,278,279]. A 2020 study found that exosomes derived from NS/PCs (isolated from fetal mouse cortex) improved functional recovery after SCI in rodents, presumably due to enhanced angiogenesis near the injury site [273]. Another preclinical study found that human iPSC-derived NS/PCs promoted functional recovery in mice with acute SCI [280]. Similarly, allogeneic NS/PCs, derived from ESCs, have been found to promote functional recovery in nonhuman primates after SCI [281].

However, results from preclinical studies have been largely inconsistent and not all studies found that reduced inflammation after acute/subacute NS/PC transplantation corresponded to gains in motor functions [282]. Differences in the NS/PC source, timing of injections, and location of injections may all affect outcomes [283,284]. For example, administration directly into, rather than adjacent to, the injury site, may result in extensive death of transplanted NS/PCs and reduced therapeutic benefit. However, one study suggested that proximity of administration and injury sites does not have an effect on transplanted NS/PC survival, as long as a threshold number of cells were delivered [284]. Thus, NS/PCs could feasibly be grafted into the lesion epicenter to avoid further damaging adjacent rostral and caudal tissues at the time of injection. Injection location concerns are at least partially mitigated by the observed migration of NS/PCs from an administration site into the injury site a few millimeters away [194,285,286].

In a chronic SCI model, transplanted human iPSC-derived NS/PCs differentiated into neurons and glia, but did not restore function [287], further indicating that one major therapeutic benefit of NS/PC transplantation is through early immunomodulation rather than through delayed regeneration. Currently, a few phase 1/2 clinical trials using ESC-derived NS/PCs are recruiting or completed with results yet to be published [216,217,218]. For example, there is an ongoing study led by Yonsei University Health System in Korea to evaluate effects of human ESC-derived NCAM+ NS/PCs, transplanted during subacute, cervical SCI [218]. There is currently one iPSC-derived NS/PC trial in Japan for subacute complete SCI that is recruiting [219]. As iPSC technology is relatively new and mostly in preclinical trials currently, we expect many more iPSC-derived NS/PCs to enter clinical trials in the near future, once production, safety, and efficacy are standardized in the preclinical setting.

#### 5.2.2. Oligodendrocyte Progenitor Cells

In contrast to multipotent NS/PCs and NS/PCs, OPCs are currently being evaluated in clinical trials as a therapy for acute SCI [221,223,288,289]. Preclinical studies in thoracic and cervical SCI models in rodents have reported that acutely administered OPCs secrete neuroprotective factors [290,291], reduce cavitation [289,292], and mediate functional recovery [289,293]. Endogenous NS/PCs in the spinal cord, specifically characterized as olig2^+^ OPCs, promote repair after SCI [294,295]. Furthermore, in rodent models, impressive motor recovery has been observed when transplanted NS/PCs differentiate into myelinating oligodendrocytes [293,296,297]. Together, these observations and the lower potential for OPCs to form tumors when compared to multipotent NS/PCs, presumably because they can be prepared as a more mature and pure population, have justified a push towards clinical translation of therapeutic OPCs [288,289].

Recently, this charge has been led by Lineage Cell Therapeutics, Inc. (Carlsbad, CA, USA), which developed a therapeutic cell line for allogeneic transplantation into the spinal cord derived from human ESCs, known as AST-OPC1. Phase 1 and 2 clinical trials conducted during the past decade in the US have aimed to evaluate the safety and efficacy of AST-OPC1 administration to patients with subacute (21–42 days following SCI) SCI (AIS A or B) [221,223]. Patients were given low level immunosuppressive therapy for 60 days after cell transplantation to prevent immunogenic rejection. GRNOPC1s, an earlier name for the AST-OPC1 lineage, showed no unanticipated serious adverse effects in 49 of 50 annual visits for the first 10 years following transplantation in thoracic patients [220], whereas AST-OPC1s resulted in 21 of 22 cervical patients recovering one or more levels of neurological function at 1-year follow up [222]. Earlier in 2021, Lineage Cell Therapeutics, Inc. announced its intention to submit an Investigational New Drug application to the FDA to set the stage for a new phase 1 clinical trial investigating AST-OPC1 delivery using Neurgain Technologies’ Parenchymal Spinal Delivery System [298], a compact device consisting of a micromanipulator and a disposable magnetic needle assembly. Neurgain’s system is expected to enable delivery of cells to the cervical spinal cord without the need to stop the patient’s respiration, provide a more accurate dosing and location of injected cells, and facilitate off-the-shelf availability of freshly thawed AST-OPC1 cells [299]. The hope is that a more precise delivery strategy may improve therapeutic efficacy to a point at which instigating a phase 3 clinical trial would be justified.

### 5.3. Glial Cells

Glia cells provide extensive trophic support to neurons in the healthy nervous system, making them good candidates for a neuroprotective therapy if transplanted during acute or subacute SCI. While we have not found any clinical trials evaluating astrocyte transplantation to date, preclinical trials have worked to identify specific populations of non-reactive astrocytes that can improve functional outcomes after SCI [300]. However, as glia originating in the CNS, which include astrocytes and oligodendrocytes, are more difficult to isolate and culture, development of glial cell therapies has been largely focused on peripherally derived glia, including olfactory ensheathing cells (OECs) and SCs that myelinate axons in the olfactory bulb and peripheral nerves, respectively. OECs have the advantage of being relatively non-invasive to isolate from a patient for autologous transplantation. OECs can also be isolated from fetal or donor organ tissues for allogenic transplantation [301,302]. While SCs can be harvested from a peripheral nerve, a neurological defect is left at the site of harvest. In addition, obtaining enough autologous SCs for transplantation from older individuals presents a significant limitation to clinical translation [303]. Alternatively, allogenic SCs can be obtained from organ donors or cadaveric tissues and cryopreserved [304,305].

#### 5.3.1. Olfactory Ensheathing Cells

OECs insulate axons in the nasal mucosa and secrete factors that support neuronal function [306]. In rodent models of SCI, acute transplantation of OECs near or into lesions has been reported to reduce inflammation, improve tissue sparing, and enhance behavioral recovery, indicating that OECs can mitigate some effects of secondary injury [307,308,309]. Alternatively, intravenously delivered OECs can traffic to SCI lesions in rats within 10 min of the injection, resulting in a reduced SCI inflammatory response, increased neurogenesis and remyelination, and improved motor function [310]. The first human trial of OEC administration for SCI evaluated patients with chronic injuries [311]. Over the past two decades, several other phase 1/2 clinical trials have evaluated transplantation of OECs from various sources, including cells derived from the SCI patient and human fetal tissues, in chronic SCI [224,225,226]. However, a meta-analysis of clinical trials in 2015 determined, while OEC transplantation was safe overall, there was no evidence to support therapeutic efficacy [312]. An ongoing clinical study is evaluating isolation of OECs from both deceased and living donors and their effects on SCI after allogenic transplantation [227]. Still, clinical trials of OEC transplantation in acute SCI have not been performed to date, possibly due to the time-intensive nature of retrieving and preparing these cells for transplantation.

#### 5.3.2. Schwann Cells

SCs are the glia responsible for myelination of axons in the peripheral nervous system (PNS) and are not normally present in the CNS. After SCI, endogenous SCs cross the compromised BSCB and enter the spinal cord, where they have been observed to remyelinate CNS axons, at least in rodents and dogs [313,314]. Preclinical studies in rodents have shown that transplantation of SCs into acute and subacute SCI reduces inflammation, improves tissue sparing, and increases axon myelination [309,315,316]. There is evidence from rodent studies that SC transplants outperform OEC and mixed SC/OEC transplants [315]. In contrast, a small clinical study reported no differences between patients receiving SCs, OECs, or SC/OEC mixtures [317]. However, all patients receiving cell transplants had improved functional recovery over patients in control groups.

As with other cell-based therapies, SCs secrete trophic factors that can promote survival and prevent “die back” of axons [318]. Neuroprotection during secondary injury has been widely proposed as a mechanism for beneficial effects of SC transplantation in SCI [315,319]. Additionally, SCs secrete ECM that is supportive of axonal growth, which may be key to their benefits. For example, human SCs transplanted into injured rodent spinal cords modify the glial scar, creating a tissue microenvironment conducive to axonal crossing [305]. As with other cell-based therapies, it is possible that acellular biological products, such as SC-derived exosomes, may provide comparable benefits to transplantation of living cells, while avoiding many of the complications associated with therapeutic cells, including reproducibility, plasticity, and immunogenicity [320].

Most clinical studies to date have transplanted autologous SCs isolated from the patient’s own sural nerve (an easily accessible sensory nerve in the lower leg) and expanded in vitro [231,321]. While allogenic cells potentially can be isolated from cadaveric tissue or potentially live donors or obtained from cultured clinical grade cell lines, use of autologous cells has the advantages of a less burdensome regulatory pathway and decreased costs [303,322]. Human trials have shown that a previous SCI does not hinder the isolation of high quality SCs from their sural nerve for sub-culture, cryopreservation, and processing for transplantation [198,323]. However, minimal rounds of sub-culturing are desirable, as expansion may reduce the myelination potential of transplanted SCs [324,325]. In a previous clinical trial, the average time from sural nerve harvest to SC transplantation in patients was around 26 days, but only yielded enough cells to allow for one round of SC transplantation [231,232].

Of note, it has been suggested that patient age may be a significant factor in determining efficacy, as SCs isolated from older individuals may exhibit variable phenotypes [303]. While SCs can be isolated from patients of all ages with similar yields of viable cells, proliferation is slower in SCs from older individuals, which may increase the time needed for expansion prior to transplantation [304,305]. A trial evaluating SC transplantation in humans was performed in Iran and published in 2008 [229]. Four patients with chronic, mid-thoracic SCI (AIS A-C) received transplants of autologous SCs isolated from sural nerve. While only one patient showed functional improvement one year after treatment, no adverse effects were found providing initial confidence in safety. In 2012, a 5-year follow-up study of six patients with chronic injuries (AIS A-C) in China confirmed the safety of transplantation of SCs, again from autologous sural nerves, and reported at least some functional improvements in all patients [230].

As with the other cell types discussed, a prevailing theory is that a major barrier to therapeutic efficacy is excessive loss of SCs after transplantation, likely through necrosis due to inflammatory or immunogenic processes [195,305,326]. Thus, researchers at The Miami Project conducted phase 1 clinical trials in the US investigating isolation of autologous SCs from sural nerves followed by transplantation into the injury epicenter using a cell injection system designed to gently deliver cells to the spinal cord [327]. Autologous SCs were transplanted in patients with subacute (within 30 days of injury) [231,232] or chronic [328,329] thoracic SCI. In the subacute study of six patients and the chronic study of eight patients, no post-transplantation adverse events or serious complications were reported at 1 year or 6–24 months, respectively. However, no strong evidence of efficacy was reported either [231,328].

## 6. Combinatorial Strategies

The consensus is that a multi-pronged approach is needed to prevent tissue loss and promote tissue regeneration in the injured spinal cord. For example, in rodent models, delivery of an immunomodulatory factor (e.g., MSSP) in combination with a neuroprotective factor (e.g., DS, growth factors, stem cells) provided additional benefits over either factor alone [330]. Moreover, several research groups have reported summative benefits of combining multiple therapeutic modalities after SCI, for example by transplanting stem cells in conjunction with small molecule drugs or biomaterial scaffolds to improve survival and promote differentiation of stem cell grafts [331]. A few small-scale clinical trials have begun to explore various combinatorial strategies to treat acute SCI. A summary of promising combinatorial strategies can be seen in Table 4.

A relatively straight-forward combinatorial approach is to perform DS at the same time as cell transplantation since the neurosurgeon may already have to open the dural/pial membranes to perform the dural DS. A phase 1/2 clinical trial in Vietnam divided acute and early subacute SCI patients within 2 weeks of injury, into two study arms: (1) patients receiving DS only and (2) patients receiving DS followed immediately by injection of autologous AD-MSCs into the intrathecal space just above and below lesions [332,333]. For patients in the treatment group, additional and escalating dosages of AD-MSCs, such that more cells were delivered with the fourth than the second injection, were administered via lumbar puncture at 30, 45, and 75 days after DS. Patients who received AD-MSCs showed improvements in all measures at the 3- and 6-month follow-up examinations when compared to before treatment. The only metric that was compared between treatment and control groups was AIS scores, which showed that AD-MSC transplantation with DS resulted in almost double the AIS improvement when compared to DS only patients. In an ongoing clinical trial in Spain, a therapeutic cell line derived from allogenic AD-MSCs (FAB117-HC) is being administered to patients within 96 h of SCI through intramedullary injection at the same time as DS [334]. A second and larger dose of FAB177-HC is then administered up to 120 h post-SCI during stabilization surgery. Importantly, this trial includes both dose escalation and efficacy studies, the latter of which have been designed to compare patients receiving therapeutic cells to those in a control group. The primary outcome measures are expected to be completed in July 2022.

Combining stem cell transplants with rehabilitation strategies, such as use of an exoskeleton, virtual reality training, or other locomotive training, has also been explored in clinical trials in the US [335,336]. Theoretically, secreted factors from therapeutic cells would act to preserve tissue during secondary injury, while rehabilitation encourages both regenerating axons and, potentially, induces new neurons differentiated from transplanted cells to make functional connections and networks. Systemic administration of drug therapies in conjunction with stem cell transplantation may also provide additive or synergistic clinical benefits. For example, phase 1/2 clinical trials in Korea have investigated treatment with both BM-MSC transplants and G-CSF [260,337]. As discussed earlier, G-CSF has positive effects on SCI, potentially including its ability to stimulate endogenous stem cells [338,339]. Clinical trials found that co-treatment with autologous BM-MSCs and G-CSF was safe [337] and led to functional benefits when administered within 2 weeks or within 3–8, but not greater than 8, weeks after SCI, indicating that these benefits arise from attenuation of secondary injury and neuroprotection rather than actual tissue regeneration [260].

Beyond co-delivery of cells and various small molecule drugs, cells can be genetically modified prior to transplantation to overproduce therapeutic biomolecules or otherwise direct cell behavior. For example, several preclinical studies have shown that transplantation of genetically engineered cells to overexpress neurotrophic factors can improve SCI outcomes over non-modified cells [340,341,342,343]. This is advantageous over direct protein or exosome delivery, which have a much shorter half-life after delivery [344]. Alternatively, stem cells can be genetically engineered to drive differentiation into a particular cell type. A recent study reported that, when transplanted acutely after SCI in a rat model, NS/PCs (isolated from E14 rat cortices) transduced to overexpress Wnt5a resulted in significant gains in functional improvement over non-transduced cells [345]. The authors suggest that these additional benefits were a result of increased neuronal differentiation of transplanted NS/PCs with Wnt5a overexpression.

**Table 4:** Clinical Studies of Combinatorial Therapeutics.

Intervention	Study Size/Type	Results	Reference
DS + Cell Transplant	N = 47 Phase 1/2	In acute and early subacute thoracic SCI patients, combination of DS and local intrathecal AD-MSCs transplantation showed improvement in all measures (AIS motor grade, electrophysiological parameters, SCI site edema, and urinary and bowel function) at 3- and 6-month follow-up examinations. AIS scores doubled in the combination group when compared to DS-only.	Tien et al. (2019) [333] NCT02034669 [332]
	Phase 1/2	Recruiting. Study ongoing.	NCT02917291 [334]
Cell Transplant + Rehabilitation	Phase 2	Recruiting. Study ongoing.	NCT03979742 [336]
Cell Transplant + Drug Therapies	N = 6 Cohort	No serious complications were found with co-treatment with autologous BM-MSCs and G-CSF in six patients with complete subacute cervical SCI.	H. C. Park et al. (2005) [337]
	N = 35 Phase 1/2	Co-treatment with autologous BM-MSCs and G-CSF led to functional benefits seemingly through attenuation of secondary injury. Administration within 2 weeks or within 3–8 weeks, but not >8 weeks showed benefits when compared to decompression alone.	Yoon et al. (2007) [260]
Biomaterial-Mediated Cell Transplant	N = 8 Phase 1	Combined application of NeuroRegen collagen scaffold and UC-MSCs is safe and feasible for clinical therapy in complete chronic cervical or thoracic SCI patients. Additionally, promising sensory-motor improvements measured at 1 year follow-up of the treatment group suggest the potential efficacy of this therapy combination.	Zhao et al. (2017) [346] NCT02352077 [347]
	N = 7 Phase 1	BM-MSC loaded NeuroRegen collagen scaffold is a safe therapy for acute thoracic SCI patients. Minimal sensory and no motor improvements were measured over a 3-year follow-up period.	W. Chen et al. (2020) [348]
	N = 40 Phase 1	Acute administration of bovine collagen scaffold loaded UC-MSCs within 21 days of injury led to improvements in AIS scores, bowel/urinary function, and injury-site electrophysiological activity over the 12-month post-operative period when compared to an intervention-free control group in cervical SCI patients.	W.S. Deng et al. (2020) [252] NCT02510365 [349]
	Phase 1/2	Recruiting. Study ongoing.	NCT03933072 [350]

Cell transplants can be genetically engineered to express multiple factors with the potential to enhance therapeutic benefits. For example, rat SCs can be engineered to secrete bifunctional neurotrophin (D15A) and chondroitinase ABC (ChABC) [351,352,353,354]. While D15A mimics effects of the neurotrophins BDNF and NT3 [355,356], ChABC is an enzyme that degrades chondroitin sulfate proteoglycans and can thus reduce glial scar deposition. In a preclinical study, transplantation of genetically engineered SCs to overexpress ChABC and D15A directly into the injury site of subacute, thoracic contusions in rats resulted in increased numbers of myelinated axons projecting into descending motor tract fibers, with corresponding improvements in motor function and reductions in thermal and mechanical allodynia [357].

As discussed in Section 5, low transplant survival rates and loss of desired phenotypes are major limitations to cell-based therapies. In preclinical studies, biomaterial scaffolds have been widely investigated as carriers for cell transplants, as detailed in a recent review article [358]. In general, biomaterials have been demonstrated to increase cell survival during [199,359] and after [200,360] injection into the spinal cord, which correlated with improved functional outcomes [361,362,363]. Furthermore, biomaterial scaffolds have been developed that can better maintain cell phenotypes of adhered cells, including MSCs [364], SCs [365], and NS/PCs [366]. Such promising preclinical results have led researchers to initiate clinical trials investigating the safety and efficacy of biomaterial-mediated cell transplantation after SCI.

Several phase 1/2 clinical trials in China have investigated transplantation of therapeutic cells, including UC-MSCs, in scaffolds derived from bovine collagen I [252,346,347,348]. One of the largest of these studies, in which treatment was administered within 21 days of cervical SCI, included two patient cohorts: (1) individuals receiving UC-MSCs on collagen-based scaffolds (N = 20) and (2) individuals receiving no additional intervention (negative control, N = 20) [252,349]. The authors found that patients who received the combinatorial treatment had improvements in AIS scores, bowel/urinary function, and injury-site electrophysiological activity at 12 months post-operation over the control group. Unfortunately, the control group did not receive DS, but they did receive other standard supportive therapies, such as infection control and rehabilitation. Additionally, this study did not provide any evidence as to whether the combinatorial therapy had benefits over either scaffolds or cell-based therapies alone.

Autologous nerve grafts, often derived from the peripheral sural nerve, represent another scaffold with potential to enhance the therapeutic effects of cell transplants after SCI. While not yet approved for clinical use, therapeutic strategies based on providing SCs or SC-laden autologous nerve grafts have been largely effective at treating PNS injuries [365,367]. In a study with a small number of patients with chronic SCI, safety and modest functional improvements were reported with transplantation of autologous sural nerve grafts and OECs [228] or BM-MSCs [368]. Clinical trials investigating transplantation of autologous OECs with sural nerve grafts are currently recruiting in Poland [350]. Alternatively, allogenic nerve grafts can be decellularized to preserve the native ECM content and structure, including aligned tubes that can guide and bundle regenerating axons, but allow for evasion of the immune response to allogenic cells [369,370]. However, while cadaver-derived, decellularized nerve grafts have been used for decades clinically in PNS repair, we have not found any reports of their transplantation into humans after SCI.

## 7. Conclusions and Future Perspectives

In summarizing clinical trials aimed at limiting secondary damage and maximizing functional recovery for patients with acute/subacute SCI, we have discussed promising therapeutic strategies in four broad categories: (1) early procedural and/or surgical management, (2) pharmacological therapies, (3) cell-based therapies, and (4) combinatorial therapies. While there are specific challenges associated with each treatment being tested (e.g., half-life limitations and off-target effects of pharmacological therapies, or time needed to expand autologous SCs within a time frame appropriate for use in acute SCI), there are also several common challenges. In particular, while several phase 2 clinical trials have been performed, only a few treatments have advanced to phase 3 clinical trials. Specific barriers to clinical translation of therapeutics discussed in this review article are highlighted below.

(1) *Recruitment of acute and subacute SCI patients*. On average, the rate of recruitment for patients with acute SCI is one patient per center per month [371,372]. This estimate can even be lower depending on the inclusion/exclusion criteria of the study and center location. Several clinical trials discussed in this study were terminated due to low enrollment. Establishing multicenter trials maybe one way to increase the number of eligible patients to be enrolled. However, multicenter trials add logistical and financial challenges. Additionally, for acute SCI patients dealing with a life changing condition, participating in a clinical trial at early timepoints after injury may be a difficult decision. Therefore, it is important that the literature provided to patients is well-developed and that designated clinical research professionals speak to patients and their families to recruit acute SCI patients into clinical trials.
(2) *Patient selection*. Patient selection is one of the most important factors that can affect the risk–benefit analysis of an intervention and efficacy of measured outcomes, but it presents a constant challenge in clinical trials. Clinical SCI is, by nature, heterogenous in its severity, location, and mode of injury. Therefore, it is essential to determine which patient populations are most likely to benefit from any therapeutic intervention. In the clinical trials reviewed here, therapeutic benefits were usually reported in around 20–30% of the patients. However, many trials included patients with AIS A–D, often with injuries at all levels of the spinal cord lumped together into a single dataset, which makes it difficult to discern whether specific injury characteristics correlated to treatment efficacy. However, this practice is not uncommon as overly restrictive patient selection criteria leads to low recruitment numbers. Therefore, care should be taken to consider all the trade-offs when making inclusion criteria for a clinical study with SCI patients. It will also be important to develop new biomarkers that can be used to stratify SCI patients, such as by evaluating the anatomical and functional deficits within the injured cord using new imaging modalities (e.g., MRI, perfusion CT, ultrasound imaging), instead of relying on neurological grading alone.
(3) *Scaling up cell production*. Challenges remain regarding the industrial scaling-up and reproducibility of bio manufactured cells for transplantation into humans [373,374]. For example, use of iPSC-derived NS/PCs would require rapid expansion and differentiation to enable administration during the acute or subacute phases of SCI. However, established culture methods used to expand and convert iPSCs to NS/PCs are primarily performed manually by highly trained technicians and, therefore, the ability to generate the large number of cells required for use in clinical therapy is limited. Furthermore, new strategies for quality control during the biomanufacturing process are needed. While previous studies have relied on a handful of biomarkers at a single time point, stem cell populations are often highly heterogenous and dynamically plastic. Thus, the field would benefit from innovative strategies to monitor the regenerative potential of therapeutic cells through dynamic measurements that better indicate their functional activity during biomanufacturing. Additionally, a better understanding of which cells within a heterogeneous population are responsible for the therapeutic benefits, identification of biomarkers for these cells, and methods for enriching populations for cells with the most therapeutic potential will all likely be required for cell-based therapies to become a standard SCI treatment.
(4) *Funding and infrastructure*. Financial and infrastructural support required to sustain large and long-term clinical trials for SCI patients are limited, in part because the population of patients suffering from SCI is relatively small when compared to patients suffering from conditions such as strokes, though both result in long-lasting neurological deficits. Therefore, more creative and non-traditional approaches, such as working with patient advocacy groups, as well as obtaining support from private foundations in addition to public institutions, are needed to support clinical studies evaluating new therapeutic strategies for patients with SCI.
(5) *Low reporting*. The lack of published results for many of the already completed clinical trials makes it difficult to draw clear conclusions about therapeutic efficacy. Of the 27 total clinical trials regarding SCIs referenced in this review, only eight were completed with publications. Five are completed with results yet to be published, nine are still recruiting, two have unknown statuses, two were terminated due to slow enrollment, and one was terminated by a business decision. Additionally, most clinical trials with published results were phase 1, and thus designed primarily to investigate treatment safety rather than efficacy.

In summary, early neuroprotective therapies offer exciting opportunities to improve the preservation and recovery of sensory and motor functions after traumatic SCI. However, logistical (patient recruiting, scaling up production of high-quality stem cells for transplantation, funding, low reporting) and pathophysiological (patient heterogeneity and lack of pathophysiological-based biomarker for recruitment) factors remain significant barriers to clinical translation of new spinal cord injury therapies. Key future steps will be to identify new biomarkers based on the pathophysiology of the injured cord to aid in the stratification of patients and to develop strategies for efficient recruiting of patients with spinal cord injuries.

## Figures and Tables

**Table 1:** Clinical Studies of Early Stage Interventions.

Intervention	Study Size/Type	Results	Reference
Therapeutic Hypothermia	N = 35	About 40% of acute cervical SCI patients demonstrated improved neurological outcomes by one ISNCSCI grade on a 10 month follow up visit for intravascular hypothermia treatment delivered 6 h following initial injury. The overall risk of thromboembolisms was low and outcomes substantiated a randomized, multi-center study.	Dididze et al. (2012) [32]
	N = 20	About 80% of patients with complete acute cervical or thoracic SCI experienced some sensory and motor gain following hypothermia treatment that occurred within 8 h of initial injury. Of fourteen patients with quadriplegia, nine experienced at least a one-grade increase in AIS grades with one patient even reaching AIS grade “D”.	Hansebout and Hansebout (2014) [33]
	N = 14	Of fourteen acute cervical SCI patients, six experienced at least a one-grade increase in AIS grades with one patient even reaching AIS grade “D”. Interestingly, embolism and coagulopathy were only noted in untreated controls, while treated patients had only respiratory and infectious complications.	Levi et al. (2010) [34]
	N = 5	While two of five acute thoracic SCI patients improved from hypothermia, three out of five had developed infections. Additionally, rewarming resulted in increased ISP, metabolic activity by cells, and pro-inflammatory factor density that, overall, worsened clinical outcomes.	Gallagher et al. (2020) [35]
	N = 41	Study completed in December 2018. [Results pending]	NCT01739010 [36]
	Randomized, Case-controlled	Recruiting. Study ongoing.	NCT02991690 [37]
Cerebrospinal Fluid Drainage	N = 22 Phase 1/2	Acute cervical or thoracic SCI patients who did not undergo CSF drainage within 48 h of injury had substantially greater intrathecal pressures compared to those measured during the intraoperative stage of treatment. Those who did receive drainage saw no change in intrathecal pressures. The use of lumbar intrathecal catheters for drainage resulted in no adverse effects.	Kwon et al. (2009) [38] NCT00135278 [39]
	N = 15 Phase 2b	Study completed in August 2019. [Results Pending]	NCT02495545 [40]
Decompression Surgery (DS)	N = 73	Thoracic or thoracolumbar SCI patients who received decompression surgery within 24 h of injury had substantially greater improvements in AIS motor scores during a 12-month follow-up as compared to those who received surgery within 24–72 h post-injury. Specifically, 24% of patients who received early surgery experienced a ≥2-grade improvement in AIS scores compared to only 5% of late surgery patients.	Haghnegahdar et al. (2020) [41]
	N = 17	Of seven acute cervical SCI patients who underwent durotomy and duroplasty, six demonstrated improvement in AIS grades, while seven of ten patients showed improvements following durotomy alone. Most notably, four of those who received durotomy and duroplasty showed two or more AIS grade improvements, whereas only one durotomy-only patient showed this same level of AIS grade improvement.	Telemacque et al. (2018) [42]
	N = 16	Durotomy followed by duroplasty lowered ISP in three of three acute cervical SCI patients who were monitored for six days following the intervention. All 16 patients showed at least one AIS grade improvement.	Zhu et al. (2019) [43]

**Table 2:** Clinical Studies of Pharmacological Therapeutics.

Therapeutic	Study Size/Type	Results	Reference
Glibenclamide	N = 3 Phase 1	Study terminated in 2021 due to principal investigator leaving university and lack of subjects for conclusive results.	NCT02524379 [125]
Minocycline	N = 52 Phase 2	Acute cervical or thoracic SCI patients received minocycline within 12 h post-injury continuing for 7 days, as well as DS within 24 h post-injury. While patients with thoracic injury saw no improvement, patients with cervical injury dramatically improved by 14 motor points, on average, compared to controls.	Casha et al. (2012) [126] NCT00559494 [127]
	Phase 3	Recruiting (last updated in 2014). Study status unknown.	NCT01828203 [128]
Riluzole	N = 36 Phase 1	Acute cervical SCI patients receiving Riluzole twice per day for fourteen days experienced robust gains in function; however, these benefits were not observed in thoracic SCI patients.	Fehlings et al. (2012) [129] Grossman et al. (2014) [130] NCT00876889 [131]
	Phase 2/3	Study terminated due to poor enrollment.	Fehlings et al. (2016) [132] NCT01597518 [133]
Sovateltide	Phase 2	Recruiting. Study ongoing.	NCT04054414 [134]
G-CSF	Phase 3	The trial showed no significant improvement in ASIA motor scores as a result of intravenously administered G-CSF compared to the placebo control group when comparing 3-, 6- and 12-month endpoints to baseline scores.	Koda et al. (2021) [135] UMIN000018752 [136]

**Table 3:** Clinical Studies of Cell-Based Therapies.

Cell Source/Type		Study Size/Type	Results	Reference
Mesenchymal Cell-Based Therapies Neuroglial Cell-Based Therapies	Adipose-Derived Mesenchymal Stem Cells	N = 10 Phase 1	Initial report of the first cervical SCI patient, who previously received acute DS, injected at 11 months after injury showed no severe adverse events at up to 18 months post-injection. Results for subsequent patients are pending.	Bydon et al. (2020) [202] NCT03308565 [203]
		Phase 2	Recruiting. Study ongoing.	NCT04520373 [204]
	Umbilical-Cord Mesenchymal Stem Cells	N = 22 Cohort	Positive response (i.e., gain in motor function, sensory abilities, regained bowel control) to UC-MSC administration in 80% of incomplete subacute or chronic cervical and/or thoracic SCI patients, but no response in complete SCI patients.	Liu et al. (2013) [205]
		N = 102 Phase 1/2	Minimal, non-lethal adverse effects (headaches, fevers, dizziness) were observed in conjunction with dramatic improvements in motor control, bowel/bladder function, and light touch sensation in subacute or chronic cervical, thoracic, or thoracolumbar SCI patients with UC-MSC transplantation.	Yang, Pang et al. (2021) [206] NCT02481440 [207]
		N = 10 Phase 1/2a	Improved pinprick sensations along the dermatomes below the injury level were observed in complete chronic SCI patients with intrathecal administration of UC-MSCs in comparison to controls.	Albu et al. (2021) [208] NCT03003364 [209]
		Phase 2	Recruiting (last updated in 2019). Study status unknown.	NCT03521336 [210]
	Bone Marrow-Derived Mesenchymal Stem Cells	N = 20 Phase 1/2	No adverse events were observed after a 2-year follow up. Majority of subacute cervical SCI patients, but only 1 of 13 chronic cervical SCI patients, showed improvement in motor and sensory performance 3 months after cell delivery.	Syková et al. (2006) [211]
		N = 9 Phase 1	No treatment-related adverse events were observed at 1-year follow up for repeated intrathecal cell injections into complete subacute or chronic thoracic SCI patients.	Satti et al. (2016) [212] NCT02482194 [213]
		N = 14 Phase 1/2	Study completed in May 2018. [Results pending.]	NCT02981576 [214]
		N = 20 Phase 1	Study completed in April 2020. [Results pending.]	NCT04288934 [215]
	Multipotent Neural Stem/Progenitor Cells and Neuronal Progenitor Cells	N = 12 Phase 1/2	Study completed in April 2015. [Results pending.]	NCT01321333 [216]
		N = 31 Phase 1/2	Terminated (2016) “based on a business decision, unrelated to any safety concerns”.	NCT02163876 [217]
		Phase 1/2	Recruiting. Study ongoing.	NCT04812431 [218]
		Phase 1	Recruiting. Study ongoing.	Sugai et al. (2021) [219]
	Oligodendrocyte Progenitor Cells	N = 5 Phase 1	No unanticipated serious adverse events were reported in 49 of 50 annual visits throughout the first 10 years following subacute transplantation into thoracic SCI patients.	McKenna et al. (2022) [220] NCT01217008 [221]
		N = 25 Phase 1/2	OPC delivery (21–42 days post-injury) in 21 of 22 cervical SCI patients resulted in recovery of one or more levels of neurological function, while 7 of 22 recovered two or more levels, in at least one side of their body at 1-year follow up.	Fessler et al. (2022) [222] NCT02302157 [223]
	Olfactory Ensheathing Cells * (* only evaluated in chronic SCI)	N = 6 Phase 1/2a	Complete chronic thoracic SCI patients received autologous cell transplants at the injury site with no observable adverse effects after 3 years. No functional improvements were observed, besides one patient displaying a gain in sensitivity to light touch and pin prick tests.	Mackay-Sim et al. (2008) [224]
		N = 11 Phase 1/2	Sensation and spasticity showed modest improvements while locomotion recovery was minimal after a 14 month-follow-up in chronic cervical SCI patients.	Wu et al. (2012) [225]
		N = 6 Phase 1	In chronic thoracic SCI patients after cell transplantation, diffusion tensor imaging revealed a reconstitution in white matter tracts for transplant patients and two patients improved from AIS grade “A” to “B” and “C”, respectively.	Tabakow et al. (2013) [226]
		Phase 1/2	Recruiting. Study ongoing.	NCT02870426 [227]
		N = 14	Motor power improvements were observed in all cases and up to four grades for two patients. However, these improvements were insufficient to enable patients to stand erect and hold their knees extended while walking unaided.	Amr et al. (2014) [228]
	Schwann Cells	N = 4 Phase 1	No adverse effects observed in chronic thoracic SCI patients with sural nerve-derived autologous SC transplants. One patient demonstrated functional improvement 1-year post-treatment, but with extensive and continuous rehabilitation.	Saberi et al. (2008) [229]
		N = 6	No adverse effects observed in chronic cervical or thoracic SCI patients who received sural nerve-derived autologous SCs. All patients demonstrated some signs of autonomic, sensory, and/or motor improvements at 5 years post-treatment.	Zhou et al. (2012) [230]
		N = 6 Phase 1	Subacute thoracic SCI patients received autologous SCs at the epicenter of the spinal lesion. No neurological or surgical complications were observed after one year.	Anderson et al. (2017) [231] NCT01739023 [232]
